# A tricyclic antidepressant, amoxapine, reduces amyloid-β generation through multiple serotonin receptor 6-mediated targets

**DOI:** 10.1038/s41598-017-04144-3

**Published:** 2017-07-10

**Authors:** Xiaohang Li, Qinying Wang, Tingting Hu, Ying Wang, Jian Zhao, Jing Lu, Gang Pei

**Affiliations:** 1 State Key Laboratory of Cell Biology, CAS Center for Excellence in Molecular Cell Science, Institute of Biochemistry and Cell Biology, Chinese Academy of Sciences, University of Chinese Academy of Sciences, 320 Yueyang Road, Shanghai, 200031 China; 2 Translational Medical Center for Stem Cell Therapy, Shanghai East Hospital, School of Medicine, Tongji University, Shanghai, China; 3 Shanghai Key Laboratory of Signaling and Disease Research, Collaborative Innovation Center for Brain Science, School of Life Sciences and Technology, Tongji University, Shanghai, 200092 China

## Abstract

Alzheimer’s disease (AD) is a major and devastating neurodegenerative disease, and the amyloid-β (Aβ) hypothesis is still the central theory for AD pathogenesis. Meanwhile, another major mental illness, depression, is one of the risk factors for AD. From a high-throughput screening (HTS), amoxapine, a typical secondary amine tricyclic antidepressant (TCA), was identified to reduce Aβ production. A follow-up investigation on antidepressants showed that most of the TCAs harbour similar activity. Previous studies have indicated that TCAs improve cognitive function in AD mouse models as well as in preliminary clinical data; however, the underlying mechanism is controversial, and the effect on Aβ is elusive. Thus, we developed a secondary screening to determine the molecular target of amoxapine, and serotonin receptor 6 (HTR6) was identified. Knockdown of HTR6 reduced the amoxapine’s effect, while the HTR6 antagonist SB258585 mimicked the activity of amoxapine. Further mechanistic study showed that amoxapine and SB258585 reduced Aβ generation through multiple HTR6-mediated targets, including β-arrestin2 and CDK5. Taken together, our study suggests that amoxapine, though no longer a first-line drug for the treatment of depression, may be beneficial for AD and further structural modification of TCAs may lead to desirable therapeutic agents to treat both AD and depression.

## Introduction

AD is the most common neurodegenerative disease and mostly affects aged cohorts, with the clinical signs and symptoms including progressive cognitive impairment and personality change^1–3^. As the hallmark of AD, the increased level of Aβ deposition closely correlates with the decline in cognitive function^4^. On the other hand, depression is also a major mental illness, and patients suffer from sadness and anxiety, eventually affecting their physical health^5^. Epidemiology studies have indicated that, as one of the most frequent comorbid psychiatric disorders in neurodegenerative diseases, depression increases the burden of care^6, 7^.

TCAs were launched into market even before the emergence of the monoamine hypothesis that explains the cause of depression^8^. Unlike the rising stars such as selective serotonin reuptake inhibitors (SSRIs), TCAs are no longer the first-line drug for depression therapy due to the complexity in their use. Interestingly, the effect of TCAs on cognition is controversial. In some reports, TCA treatment worsened the cognition^9, 10^; however, other studies have demonstrated that there could be some improvement in cognitive function^11, 12^. In the meantime, significant improving effects of TCAs on cognitive function in AD animal models have also been recently described by different groups^13–15^, whereas their effect on Aβ generation is inconclusive^13^. Researchers have also noticed that imipramine facilitates secreted amyloid precursor protein (sAPP) generation in primary cultured rat neurons^16^. In addition, protriptyline has been reported to bind and inhibit β-site amyloid precursor protein cleaving enzyme 1 (BACE1) activity in an *in silico* screening^17^. All of these pieces of evidence indicate that TCAs may improve AD symptoms by somehow modulating APP processing. Here, following our HTS data, we have performed cellular experiments to determine the molecular mechanism of TCA’s action on Aβ generation.

## Results

### High-throughput screening identifies amoxapine as an Aβ-reducing agent

**Figure 1:**
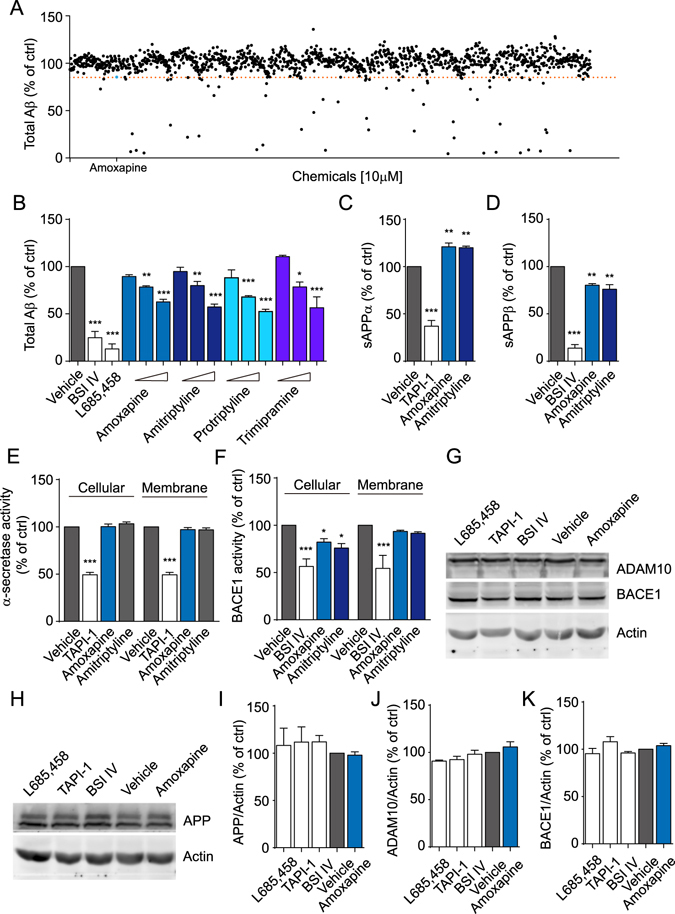
Amoxapine, a typical secondary amine TCA, reduces Aβ generation in a dose-dependent manner. (**A**) Representative results of the screening for chemicals that reduce Aβ generation. Two hours after cell seeding, HEK293-APPsw cells were treated with 10 μM of chemicals for 24 hours, and the Aβ concentration in the supernatant was measured by ELISA. Amoxapine is highlighted in blue. (**B**) The levels of Aβ produced by SK-N-SH cells in response to vehicle (0.1% DMSO), 10 μM BACE inhibitor IV (BSI IV), 10 μM L685,458, or the indicated compounds at 1 μM, 3 μM or 10 μM for 24 hours. (**C**,**D**) The levels of sAPPα (**C**) and sAPPβ (**D**) produced by SK-N-SH cells in response to vehicle (0.1% DMSO), 100 μM TAPI-1, 10 μM BSI IV, or the indicated compounds at 10 μM for 24 hours. (**E**,**F**) The measurements of α-secretase (**E**) and BACE1 (**F**) activity by fluorogenic substrate assay after treatment with vehicle (0.1% DMSO), 100 μM TAPI-1, 10 μM BSI IV, or 10 μM of the indicated chemicals. For the cellular treatment, SK-N-SH cells were treated with the indicated compounds for 24 hours before designated to the fluorogenic substrate assay. For the membrane treatment, the membrane fraction of SK-N-SH cells was prepared before compound treatment. (**G**) Representative image of a western blot showing the expression of α-secretase (ADAM10) and BACE1 after treatment with vehicle (0.1% DMSO), 100 μM TAPI-1, 10 μM BSI IV, 10 μM L685,458, or 10 μM amoxapine for 24 hours. Actin was used as loading control. (**H**) Representative image of a western blot showing the expression of APP after treatment with vehicle (0.1% DMSO), 100 μM TAPI-1, 10 μM BSI IV, 10 μM L685,458, or 10 μM amoxapine for 24 hours. Actin was used as a loading control. (**I**,**K**) The statistical analysis of G and H using ImageJ. Data are presented as the mean ± s.e.m. *p < 0.05, **p < 0.01 and ***p < 0.001 compared to the control of each group. One-way ANOVA with post hoc comparison test (**B**–**F** and **I**,**K**).

A commercially available chemical library composed of 1280 pharmacologically active compounds was assigned to the high-throughput Aβ screening using a sandwich Enzyme-Linked ImmunoSorbent Assay (ELISA). In HEK293 cells stably expressing APP Swedish mutant (referred to as HEK293-APPsw), 69 chemicals at 10 μM showed the ability to reduce the extracellular Aβ amount (≥20%), and among them, amoxapine suppressed the Aβ level by approximately 20% (Fig. 1A) without influencing cell viability (data not shown). We then validated the data in SK-N-SH, a human neuronal cell line. As the direct inhibition of the secretases of the amyloidogenic pathway leads to the decrease in Aβ generation, 10 μM of a BACE1 inhibitor, BACE inhibitor IV (BSI IV) and 10 μM of a γ-secretase inhibitor, L685,458, were used as positive controls. Amoxapine dose-dependently reduced the amount of Aβ secreted into the medium, reaching 37.32 ± 2.75% (mean ± s.e.m.) reduction at 10 μM without affecting cell viability (Fig. 1B and Supplementary Figure 1A). Amoxapine is a secondary amine tricyclic antidepressant and was approved for treating major depressive disorder in the US in 1992^18^. It was curious to us whether other tricyclic antidepressants harbour similar activity towards Aβ generation. In SK-N-SH cells, amitriptyline, protriptyline and trimipramine also dose-dependently suppressed extracellular Aβ levels with no obvious cytotoxicity (Fig. 1B and Supplementary Figure 1A). Since TCAs are no longer the first-line drug for depression therapy, we also tested another major type of antidepressants that is currently in use, the SSRIs, in the same system. Compatible with the *in vivo* data of previous reports^19–21^, 10 μM of citalopram, 10 μM of fluoxetine and 3 μM of sertraline slightly reduced cellular Aβ generation with 16.57 ± 2.89%, 25.95 ± 2.92%, and 27.32 ± 2.72%, respectively, without cytotoxicity (Sup. Fig. 1B,C). We further investigated the effect of amoxapine on the generation of two major Aβ species, Aβ40 and Aβ42. The data showed that amoxapine reduced both species of Aβ with similar potency (Sup. Fig. 1D,E), indicating that amoxapine did not work as a gamma secretase modulator (GSM). We then monitored the extracellular sAPPα and sAPPβ levels by using ELISA (Fig. 1C and D). TAPI-1 is an α-secretase inhibitor and, consistent with previous reports, significantly reduced the extracellular sAPPα level at 100 μM, while BSI IV (10 μM) significantly reduced the extracellular sAPPβ level^22, 23^. In addition to the alteration in the total extracellular Aβ level, the sAPPα level increased, and the sAPPβ level decreased with amoxapine (10 μM) or amitriptyline (10 μM) treatment (sAPPα-amoxapine: 121.10 ± 3.87%, sAPPα-amitriptyline: 120.00 ± 1.61%; sAPPβ-amoxapine: 80.30 ± 1.76%, sAPPβ-amitriptyline: 76.04 ± 4.87%), indicating that an activity shift occurs between ADAM10 and BACE1. By using a fluorogenic substrate secretase activity assay, we determined the compounds’ effect on the activity of α-secretase or BACE1. The enzymatic activity of α-secretase and BACE1 was significantly inhibited by TAPI-1 (100 μM) or BSI IV (10 μM) treatment (Fig. 1E). For amoxapine and amitriptyline, α-secretase activity remained unchanged with cellular (i.e., application of 10 μM of amoxapine or amitriptyline to the cell before membrane extraction) or *in vitro* (i.e., application of 10 μM of amoxapine or amitriptyline to the extracted membrane fraction) treatment (Fig. 1E). Interestingly, BACE1 activity was significantly reduced with cellular amoxapine (82.128 ± 3.64%) or amitriptyline (75.992 ± 4.68%) treatment, while it remained unchanged with *in vitro* treatment (Fig. 1F). We then examined whether the protein level of ADAM10, BACE1 or full-length APP was modulated by amoxapine (10 μM) using western blot analysis. As shown in Fig. 1G–K, all expression levels were intact. All of these data suggest that amoxapine reduces Aβ generation possibly through indirect modulation of BACE1 activity.

### Amoxapine reduces Aβ generation through HTR6

**Figure 2:**
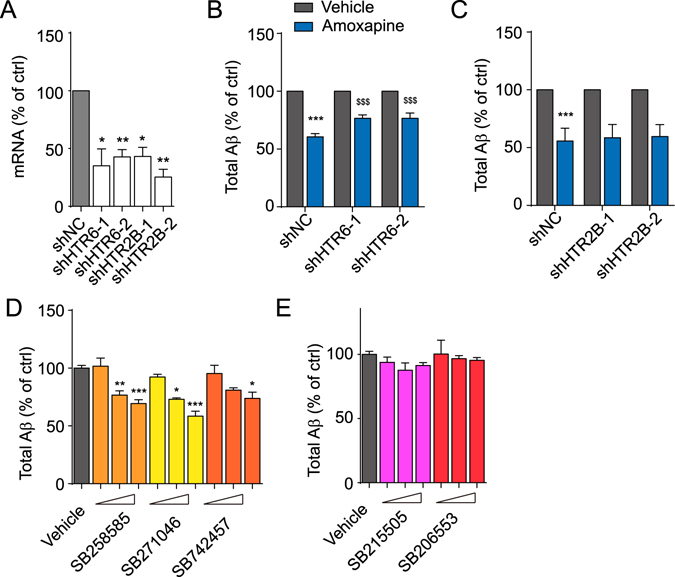
Serotonin receptor 6 mediates the effect of amoxapine. (**A**) The mRNA level of HTR6 and HTR2B in SK-N-SH cells with the infection of scrambled, HTR6 or HTR2B gene-specific shRNA. (**B**,**C**) The levels of Aβ produced by SK-N-SH cells after treatment with vehicle (0.1% DMSO) or amoxapine at 10 μM for 24 hours in the cells infected as described in (**A**). (**D**) The levels of Aβ produced by SK-N-SH cells in response to the indicated HTR6 antagonists at 1 μM, 3 μM or 10 μM for 24 hours. (**E**) The levels of Aβ produced by SK-N-SH cells in response to the indicated HTR2B antagonists at 1 μM, 3 μM or 10 μM for 24 hours. Data are presented as the mean ± s.e.m. *p < 0.05, **p < 0.01 and ***p < 0.001 compared to the control of each group or the control of the shNC group. ^$^p < 0.05, ^$$^p < 0.01 and ^$$$^p < 0.001 compared to amoxapine of the shNC group. One-way ANOVA with post hoc comparison test (**A**,**D** and **E**) and two-way ANOVA with post hoc comparison test (**B**,**C**).

Amoxapine and other TCAs are antagonists of dozens of G-protein-coupled receptors (GPCRs). To sort out the potential target(s) responsible for the Aβ-reducing activity of amoxapine, we first monitored the expression profile of amoxapine-targeted molecules in SK-N-SH cells. Amoxapine is mainly regarded as an antagonist of several dopamine receptors and serotonin receptors^24^. The most abundant subtypes of dopamine receptors and serotonin receptors expressed in SK-N-SH cells are DRD2, HTR6, HTR2B and HTR4 (Sup. Fig. 2A). Thus, we created a pool of shRNAs to knockdown individual GPCRs. Packaged as lentivirus, equal titers of specific shRNAs were applied to SK-N-SH cells. The knockdown efficiencies of the shRNAs were determined by quantitative RT-PCR. As shown in Fig. 2A and Supplementary Figure 2B, mRNA levels of these GPCRs were reduced by 60.361 ± 3.30% (shDRD2), 64.905 ± 14.56% (shHTR6-1), 57.158 ± 6.28% (shHTR6-2), 56.76 ± 7.85% (shHTR2B-1), 74.66 ± 6.77% (shHTR2B-2), 60.366 ± 6.36% (shHTR4), and 51.267 ± 2.48% (shHTR7), respectively, 72 hours post infection (h.p.i.). Then, we tested which receptor is responsible for the effect of amoxapine. We treated SK-N-SH cells with amoxapine 72 h.p.i. Twenty-four hours later, the supernatant was collected, and the Aβ within was detected by ELISA. The knockdown of HTR6 significantly reduced the activity of amoxapine in its suppression of Aβ generation (from 39.28 ± 2.77% with shNC to 23.441 ± 3.03% with shHTR6-1 and 23.432 ± 4.64% with shHTR6-2) (Fig. 2B), while none of the other three GPCRs showed a similar property (Fig. 2C and Supplementary Figure 2C–E), suggesting that amoxapine may function through HTR6. We then asked whether specific HTR6 antagonists mimic amoxapine’s efficacy for reducing Aβ production. SB258585, SB271046 and SB742457 are selective antagonists of HTR6 with a Ki of approximately 10 nM, while SB215505 and SB206553 are potent and selective HTR2B/HTR2C antagonists^25^. We applied those compounds to SK-N-SH cells and detected the Aβ level in the medium 24 hours after treatment. Consistent with the effect of amoxapine, all three HTR6 antagonists reduced Aβ generation dose-dependently without an obvious effect on cell viability (Fig. 2D and Supplementary Figure 2,F); however, HTR2B/HTR2C antagonists treatment did not change the extracellular Aβ level (Fig. 2E). Collectively, our data indicate that HTR6 mediated amoxapine’s effect on Aβ generation.

### Amoxapine and SB258585 reduce Aβ generation in human neuronal differentiated NSCs

**Figure 3:**
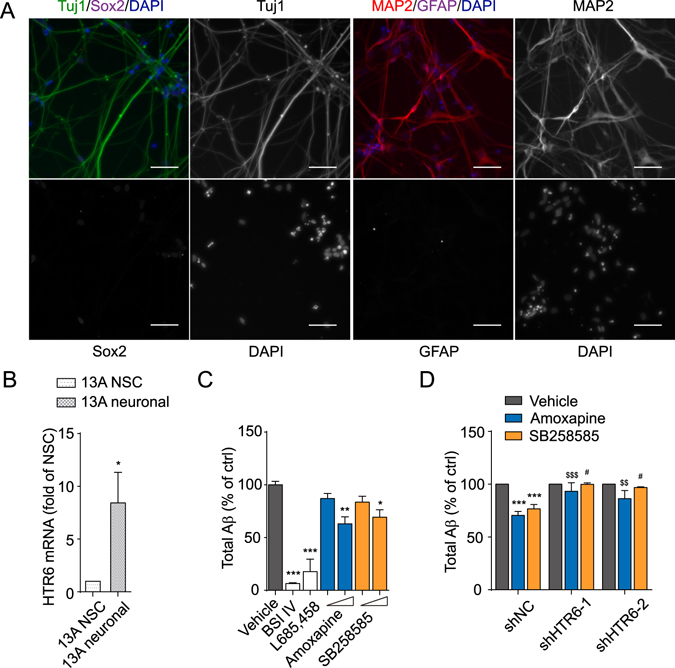
Amoxapine modulates Aβ generation in human neuronal cells differentiated from NSCs. (**A**) Representative image of neuronal differentiated human neural stem cells (NSCs) stained with Tuj1, Sox2, MAP2, GFAP or DAPI. Scale bar, 50 μm. (**B**) The mRNA level of HTR6 in human NSCs and NSC differentiated neuronal cells. (**C**) The levels of Aβ produced by neuronal differentiated NSCs in response to vehicle (0.1% DMSO), 10 μM BSI IV, 10 μM L685,458, or the indicated compounds at 3 μM or 10 μM for 24 hours. (**D**) The levels of Aβ produced by neuronal differentiated NSCs after treatment with vehicle (0.1% DMSO), amoxapine or SB258585 at 10 μM in the cells with the infection of scrambled or HTR6 gene-specific shRNA. Data are presented as the mean ± s.e.m. *p < 0.05, **p < 0.01 and ***p < 0.001 compared to the control of each group or the control of shNC group. ^$^p < 0.05, ^$$^p < 0.01 and ^$$$^p < 0.001 compared to amoxapine of the shNC group. ^#^p < 0.05, ^##^p < 0.01 and ^###^p < 0.001 compared to SB258585 of the shNC group. Two-tailed t-test (**B**), one-way ANOVA with *post hoc* comparison test (**C**) and two-way ANOVA with *post hoc* comparison test (**D**).

To confirm the activity of amoxapine in a more relevant system, we used neuronal differentiated human iPSC-derived NSCs as a model, which were primarily taken as fibroblasts from people. The neuronal differentiated cells were evaluated for their neuronal linage property by immunostaining and quantitative RT-PCR. Most of the differentiated cells were positive for Tuj1 and Map2, markers for neurons, and negative for Sox2, a marker for NSCs (Fig. 3A). Furthermore, the transcription level of Nestin and Sox2 was significantly down-regulated in the induced neuronal cells (Sup. Fig. 3A), and meanwhile, Doublecortin (DCX), β-III tubulin (Tubb3), microtubule-associated protein 2 (MAP2), NeuroD1, synapsin 1 (SYN1), neural cell adhesion molecule (NCAM), and microtubule-associated protein Tau (MAPT) were all up-regulated (Sup. Fig. 3B), indicating the formation of neurons in the cell population. Interestingly, the genes encoding HTR6 and the GABAergic neuronal marker, vGAD67, were also up-regulated after the three-week differentiation (Fig. 3B and Supplementary Figure 3,C). Using this neuronal cell model, we investigated the effect of amoxapine and SB258585 on Aβ generation. BSI IV and L685,458 again served as positive controls and significantly inhibited Aβ generation. In these cells, amoxapine and SB258585 consistently reduced Aβ generation at 10 μM by 36.97 ± 6.61% and 30.64 ± 6.98% (Fig. 3C). In addition, the knockdown of HTR6 diminished such action of those compounds (for amoxapine: from 29.609 ± 3.74% with shNC to 6.69 ± 8.16% with shHTR6-1 and 13.653 ± 7.73% with shHTR6-2; for SB258585: from 23.357 ± 4.22% with shNC to 0.11 ± 1.44% with shHTR6-1 and 3.043 ± 0.654% with shHTR6-2) (Fig. 3D). Taken together, our results indicate that amoxapine and SB258585 reduced Aβ generation through HTR6 in human neuronal differentiated NSCs.

### Amoxapine and SB28585 reduce Aβ generation through HTR6-mediated multi-targets

**Figure 4:**
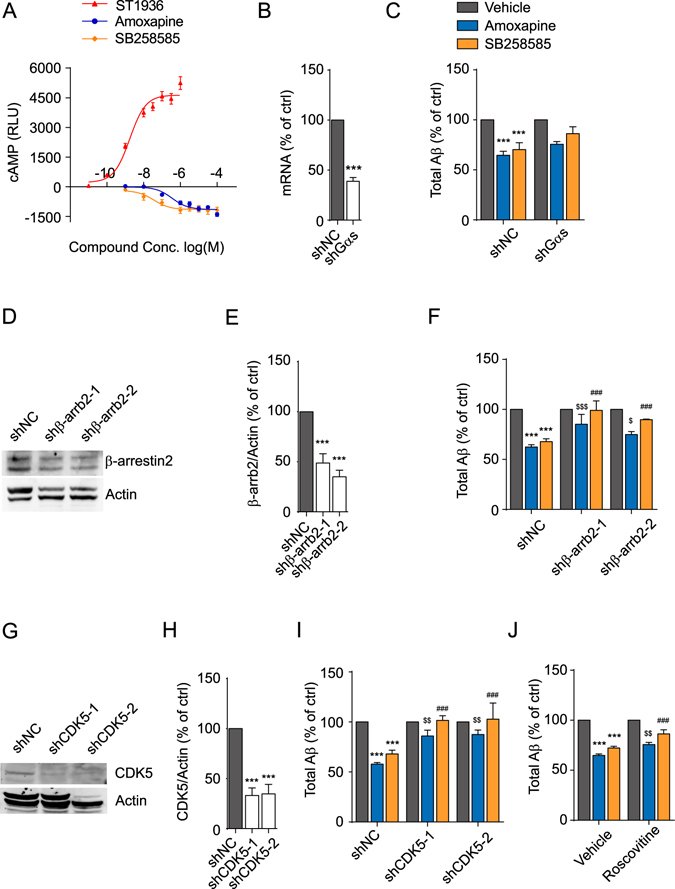
Amoxapine reduces Aβ generation through multiple HTR6-mediated targets. (**A**) The cAMP responses after stimulation with the indicated compounds at the indicated concentrations in SK-N-SH cells infected with HTR6 lentivirus. (**B**) The mRNA level of Gα_s_ in SK-N-SH cells with the infection of scrambled or Gα_s_ gene-specific shRNA. (**C**) The levels of Aβ produced by SK-N-SH cells after treatment with vehicle (0.1% DMSO), amoxapine or SB258585 at 10 μM for 24 hours in the cells infected as described in (**B**). (**D**) Representative image of a western blot showing the expression of β-arrestin2 in SK-N-SH cells with the infection of scrambled or β-arrestin2 gene-specific shRNA. Actin was used as loading control. (**E**) The statistical analysis of **D** using ImageJ. (**F**) The levels of Aβ produced by SK-N-SH cells after treatment with vehicle (0.1% DMSO), amoxapine or SB258585 at 10 μM for 24 hours in the cells infected as described in (**D**). (**G**) Representative image of a western blot showing the expression of CDK5 in SK-N-SH cells with the infection of scrambled or CDK5 gene-specific shRNA. Actin was used as loading control. (**H**) The statistical analysis of (**G**) using ImageJ. (**I**) The levels of Aβ produced by SK-N-SH cells after treatment with vehicle (0.1% DMSO), amoxapine or SB258585 at 10 μM for 24 hours in the cells infected as described in (**G**). (**J**) The levels of Aβ produced by SK-N-SH cells after treatment with vehicle (0.1% DMSO), amoxapine or SB258585 at 10 μM for 24 hours in SK-N-SH cells with 45 min pretreatment with 50 μM of the CDK5 inhibitor roscovitine. Data are presented as the mean ± s.e.m. *p < 0.05, **p < 0.01 and ***p < 0.001 compared to the control of each group or the control of shNC group. ^$^p < 0.05, ^$$^p < 0.01 and ^$$$^p < 0.001 compared to amoxapine of the shNC group. ^#^p < 0.05, ^##^p < 0.01 and ^###^p < 0.001 compared to SB258585 of the shNC group. Two-tailed t-test (**B**), one-way ANOVA with *post hoc* comparison test (**E** and **H**), and two-way ANOVA with *post hoc* comparison test (**C**,**F**,**I** and **J**).

HTR6 is a constitutively active Gα_s_-coupled receptor. Using a GloSensor^TM^ cAMP assay, we monitored the dynamic change of the intracellular cAMP level in response to the compounds being studied. ST1936 is a high-affinity HTR6-specific agonist with a ki of 13 nM^26^. As previously reported, ST1936 stimulated cAMP production with an EC_50_ of 1.7 nM, indicating that the experiment works (Fig. 4A). Consistent with the previous reports that amoxapine and SB258585 are antagonists of HTR6, suppressed cAMP levels were observed with treatment with these compounds with an IC_50_ of 0.3 μM and 30 nM, respectively (Fig. 4A). As cAMP can modulate Aβ production, we examined whether amoxapine-mediated cAMP signalling is responsible for the change in Aβ production by knocking-down Gα_s_. The knockdown efficiency of the shRNAs targeting Gα_s_ was determined by quantitative RT-PCR. As shown in Fig. 4B, the mRNA level of Gα_s_ was reduced by 61.08 ± 3.80% 72 h.p.i. Under such conditions, the Aβ-reducing effect of the compounds still subsisted (Fig. 4C), suggesting that Gα_s_-independent pathways may be the major contributor. HTR6 also mediates non-canonical β-arrestin2-dependent signalling^27, 28^. Therefore, we tested whether β-arrestin2 plays a part in the compounds’ effect. β-arrestin2-targeted-shRNA-containing lentiviruses (shβ-arrb2-1 and shβ-arrb2-2) were applied to the SK-N-SH cells. As shown in Fig. 4D and E, the protein level of β-arrestin2 was significantly diminished (51.14 ± 9.24% with shβ-arrb2-1 and 64.91 ± 6.48% with shβ-arrb2-2) 72 h.p.i. Under such conditions, the effect of amoxapine and SB258585 was greatly reduced (for amoxapine: from 37.568 ± 2.25% with shNC to 14.772 ± 9.775% with shβ-arrb2-1 and 25.031 ± 3.03% with shβ-arrb2-2; for SB258585: from 32.141 ± 2.87% with shNC to 0.891 ± 9.49% with shβ-arrb2-1 and 10.217 ± 0.588% with shβ-arrb2-2) (Fig. 4F), indicating that amoxapine and SB258585 suppress Aβ generation in a β-arrestin2-dependent manner. SB258585 was known to interfere with the interaction of HTR6 and CDK5, which subsequently down-regulates CDK5 activity^29^. As a result, we hypothesized that CDK5 may also be part of the mechanism. To test this, we applied CDK5-targeted shRNA-containing lentiviruses (shCDK5-1 and shCDK5-2). The protein level of CDK5 was markedly reduced (66.86 ± 7.54% with shCDK5-1 and 65.15 ± 9.45% with shCDK5-2) 72 h.p.i. as monitored by western blot (Fig. 4G and H). The knockdown of CDK5 also significantly attenuated the compounds’ effect (for amoxapine: from 42.24 ± 1.69% with shNC to 14.012 ± 5.79% with shCDK5-1 and 12.606 ± 4.61% with shCDK5-2; for SB258585: from 31.976 ± 3.77% with shNC to −1.506 ± 4.642% with shCDK5-1 and −2.801 ± 16.141% with shCDK5-2) (Fig. 4I). In addition, the chemical blockage of CDK5 using roscovitine also significantly compromised the compounds’ effect (for amoxapine: from 35.143 ± 1.51% with vehicle to 24.351 ± 2.11% with roscovitine; for SB258585: from 27.770 ± 1.65% with vehicle to 13.719 ± 4.22% with roscovitine) (Fig. 4J). Taken together, our data suggest that amoxapine may modulate Aβ generation in a HTR6-mediated multiple target-dependent manner.

## Discussion

After being first marketed in the 1950s, TCAs have long been in clinical use as major anti-depressants for at least three decades. Nevertheless, the severe side effects have TCAs have cast a shadow ever since their development, and resulting in their gradual replacement by SSRIs or other more specific anti-depressive agents gradually in the late 1990s. As every cloud has a silver lining, there are scattered clinical data and several AD animal model results suggesting that TCAs may potentially improve AD symptoms through multiple mechanisms^11–15^. This study unintentionally identified that a typical secondary amine TCA, amoxapine, clearly reduced cellular Aβ generation in an Aβ-targeted high-throughput screening, which was further verified in several cell models. Though we have suggested that HTR6 is a major target for its Aβ-reducing effect, dirty drugs such as TCAs may exert their effect on the central nervous system (CNS) through not only HTR6 but also other GPCRs, transporters or unknown targets. Apart from the mechanism proposed in this study, we are still working on other possibilities that may be involved.

In the CNS, HTR6 is mainly expressed in neurons of limbic regions including the striatum, cortex and hippocampus and plays a role in regulating cognitive function including memory and mood^30, 31^. Revealed by a number of animal behaviour studies, the knockdown or antagonism of HTR6 attenuate the memory deficits in AD animal model and shows anti-depressive activity^32–34^. Based on our secondary screening data, HTR6 is responsible for the Aβ-reducing activity of amoxapine, suggesting that compounds targeting HTR6 may modulate both cognition and Aβ generation, which directly fine-tunes AD pathogenesis. Additionally, three HTR6 antagonists tested in our experiment were also effective at reducing Aβ generation, although they showed differences in efficacy (Fig. 2D). Interestingly, among those antagonists, two compounds entering clinical phase III trials reduced Aβ generation to a smaller extent than SB271046, which had failed in a phase I clinical trial due to its poor ability to penetrate into the CNS after systemic dosing^35, 36^. Considering the recent failure of idalopirdine in a phase III clinical trial, our data suggests that the Aβ-reducing activity may be worth considering when researchers design and modify the HTR6 antagonists for the development of AD drugs. However, whether HTR6 is a perfect molecular target against AD may need further investigation. In addition, SSRIs were less effective at reducing Aβ generation in the same cellular model compared with TCAs, although a minor but significant *in vivo* efficacy has been observed and reported by several groups^19, 20^. All of these results indicate that compounds interfering with serotonin signalling may have an impact on Aβ generation.

In principle, HTR6 is a Gα_s_-coupled receptor with constitutive activity, and the decreased level of cAMP upon antagonist treatment was obviously correlated with reduced Aβ production, though it may not be the whole story^37^. In AD, there is mounting evidence implicating the participation of GPCRs in the modulation of APP processing and cognitive function. As reported by Ni. *et al*., Teng. *et al*. and Thathiah. *et al*., β2 adrenergic receptor (β2AR), δ-opioid receptor (DOR) and G-protein coupled receptor 3 (GPR3) regulate γ-secretase activity by direct interaction with secretases or via the assistance of β-arrestin2, which finally leads to a change in Aβ generation^38–40^. On the other hand, the angiotensin II receptor type 2 (AT2R) and HTR6 modulate AD pathogenesis by regulating glutamate and/or acetylcholine signalling^35, 41–43^. In our study, amoxapine modulated Aβ generation through targeting HTR6 in a β-arrestin2-dependent manner, as the knockdown of β-arrestin2 reduced amoxapine’s effect (Fig. 4F). In addition, the downstream molecules of HTR6, including CDK5, may also contribute to the amoxapine’s effect (Fig. 4I and J). Our data indicates that compounds antagonizing HTR6 signalling may harbour disease-modifying activity and be beneficial for AD.

In spite of the numerous hypotheses that researchers have established to explain the aetiology and pathogenesis of psychiatric disorders such as depression and neurodegenerative diseases such as AD, the upstream determinants and downstream effects for those diseases always overlap, which indicates that drugs targeting one disease may bring some other effect to the CNS either directly or indirectly^44^. In our previous studies, anti-Parkinson’s disease (PD) drugs, istradefylline, levodopa and piribedil, were all found to promote Aβ generation^45, 46^. By either modulating the interaction between the adenosine A_2A_ receptor (A_2A_R) and γ-secretase or regulating the D_2_R-mediated β-arrestin2-dependent signalling pathway, those anti-PD drugs may contribute to AD-like pathology. Conversely, we reported here that a group of TCAs harbour the Aβ-reducing activity. Our data suggest that a multi-target-directed ligand may introduce crosstalk between different neurological diseases and it is necessary to evaluate the potency of therapeutic candidates in the pipeline using multiple neurological disease models.

## Materials and Methods

### Compounds, Reagents, and Antibodies

Fluoxetine, TAPI-1, SB271046, SB742457, L-685,458 and roscovitine were purchased from Selleck Chemicals. Amitriptyline hydrochloride, ST1936 and SB258585 were from Tocris Bioscience. Protriptyline hydrochloride, amoxapine, trimipramine maleate, SB215505, SB206553, cAMP, L-ascorbic acid and DAPI were purchased from Sigma. Escitalopram oxalate was purchased from Lundbeck. Sertraline hydrochloride was from Pfizer. BACE inhibitor IV (BSI IV) was from Calbiochem. Recombinant human BDNF, GNDF, and IGF-I were from Peprotech. CellTiter-Glo was from Promega. Immunoblotting was performed with the following antibodies: anti-ADAM10 (Ab1997, Abcam), anti-BACE1 N-termimus (AP7774b, Abgent), anti-APP-CTF (A8717, Sigma), anti-actin (A2066, Sigma), anti-CDK5 (sc-173, Santa Cruz), and Rabbit anti-β-arrestin-1/2 (A1CT) antibody was a kind gift from Dr. Robert J. Lefkowitz. Immunofluorescence staining was performed with the following primary antibodies: anti-Tuj1 (801201, BioLegend), anti-Sox2 (sc-17320, Santa Cruz), anti-Map2 (Ab5622, Millipore), and anti-GFAP (sc-6170, Santa Cruz).

### Cell Culture, Plasmids, and shRNA

SK-N-SH cells and HEK293 cells were purchased from ATCC. HEK293/APPswe cells were transfected, selected with antibiotics (G418, 1 mg/ml), and maintained in lab. All of these cell lines were cultured in Modified Eagle’s Medium (MEM) with 10% (w/v) heat-inactivated foetal bovine serum (FBS) in a humidified incubator with 5% CO2/95% air (v/v) at 37 °C. Human iPSC-derived NSCs were provided by IxCell Biotechnology., Ltd. and maintained with neural stem cell culturing basal medium containing 1:50 NSC supplement (I × Cell).

The human HA-tagged HTR6 construct was kindly provided by Dr. Xie Xin (SIMM, China). The viral constructs encoding the HT6R or F20 were generated by subcloning the cDNA into a FUGW vector using the BamHI restriction sites. The constructs were verified by sequencing. The primers used are listed in Supplementary Table 1.

For RNA interference (RNAi) experiments, shRNA targeting human HTR6, HTR2B, HTR4, HTR7, DRD2, Gα_s_, ARRB2 or CDK5 was cloned into a pLKO.1 vector following the online protocol (Addgene, http://www.addgene.org/tools/protocols/plko/). All targeting sequences are listed in Suppplementary Table 2. A pLKO.1-sh-SCRAM vector expressing a scrambled sequence complementary to no human gene was used as a control.

### Lentiviral Constructs and Infection

Human embryonic kidney 293T (HEK293T) cells were seeded at a density of 7.5 × 10^6^ cells in 100-mm dishes. On the following day, cells were transfected with 20 μg of shRNA constructs, 16 μg of pSPAX2, and 6 μg of pMD2G. The transfection was routinely performed by using the calcium phosphate transfection method. Cells were allowed to produce lentivirus for 48 hours. The virus-containing supernatant was collected and then centrifuged at 1000 × g for 5 min, before being passed through 0.45-μm filters. The lentiviruses were further concentrated by ultracentrifugation at 27,000 × g for 2 hours. The pellets were then resuspended in 200 μl of PBS, aliquoted and stored at −80 °C. The virus titers are determined by flow cytometry (FACS) analysis. For knockdown experiments, SH-N-SK cells or neuronal differentiated NSCs were seeded in 100-mm dishes or 24-well plates before concentrated lentiviruses infection (minimum multiplicity of infection) in the presence of Polybrene (Sigma, 8 μg/ml). After 24 hours, the medium was refreshed. The efficiency of the shRNA was determined by quantitative RT-PCR or western blot at 72 or 96 hours post infection (h.p.i.).

### ELISA for Aβ, sAPPα and sAPPβ

HEK293/APPswe cells, SK-N-SH cells, and induced human neuronal cells were treated with chemicals at the indicated concentrations for 24 h. The conditioned medium was then collected and subjected to a sandwich ELISA for the measurement of total Aβ level. The measurement was done according to the manufacturer’s guidelines. ELISA kits for total human Aβ were obtained from ExCell Bio. ELISA kits for human sAPPα or sAPPβ were obtained from IBL.

### Differentiation of Neuronal Cells from Human NSCs

The differentiation of NSC into neuronal cells was performed according to the previous reports^47, 48^ with minor modification. In detail, NSC 13A cells were detached by accutase and resuspended in neural stem cell culturing basal medium containing 1:50 NSC supplement (IxCell). Then, 3 × 10^4^ 13A cells per well were seeded in 24-well plates coated with laminin (Sigma). On the second day, the medium was changed to neuron differentiation medium (Neurobasal, 1 × B27, 1 × N2, 100 nM cAMP, 1 μg/ml L-ascorbic acid, 10 ng/ml BDNF, 10 ng/ml GDNF and 10 ng/ml IGF-I). The medium was refreshed every two days.

### Cell Viability Measurement

Chemical-treated SK-N-SH cells were subjected to the CellTiter-Glo Luminescent Cell Viability Assay (Promega) following the manufacturer’s instructions.

### cAMP Assay

Intracellular cAMP was measured using a GloSensor^TM^ cAMP assay following the manufacturer’s instruction with minor modification (Promega). SK-N-SH cells were infected with HTR6 and F20-packaging lentivirus and seeded in white 96-well plates (Costar). Before the cAMP assay, the medium was removed and replaced with fresh medium containing 2% (v/v) GloSensor^TM^ cAMP reagent. After 90 min incubation at 37 °C, cells were equilibrated at room temperature (RT) for 15 min and treated with the ligands at the indicated concentrations for another 15 min followed by the measurement of luciferase activity.

### Immunofluorescence Microscopy

The induced human neuronal cells grown on a coverslip were fixed with 4% paraformaldehyde (PFA) in PBS for 20 min. Cells were permeabilized and blocked with PBS/0.2% Triton X-100/1% BSA for 30 min followed by incubation with the indicated primary antibodies for 2 hours at RT. After washing with PBS/1% BSA three times, cells were incubated with Alexa Fluor 647-labelled donkey anti-goat IgG, Alexa Fluor 594-labelled donkey anti-rabbit IgG or Alexa Fluor 488-labelled donkey anti-mouse secondary antibodies in the dark for 1 hour, washed with PBS/1% BSA, stained with DAPI (1 μg/ml, 10 min), and mounted on slides. Images were acquired using CellInsight CX7 (Thermo Fisher Scientific) with a 20×/0.40 NA objective (Olympus).

### Reverse Transcription and Quantitative Real-Time PCR

Total RNA was extracted with TRI Reagent (T9424; Sigma) according to the manufacturer’s instructions. Random hexamer primers and MMLV Reverse Transcriptase (M5301; Promega) were used for reverse transcription. All gene transcripts were quantified by quantitative real-time PCR performed with a 2 × HotStart SYBR Green qPCR Master Mix (ExCell Bio, Shanghai, China) on a Stratagene Mx3000P (Agilent Technologies). The primers used for the detection of mRNA levels of human genes are listed in Supplementary Table 3. All the primers were synthesized and purified by Shanghai Sunny Biotechnology Co., Ltd.

### Statistical Analysis

All experiments were repeated at least three times. Data are representative or mean ± s.e.m. All data were analyzed by Prism 6.0 (GraphPad Software Inc., San Diego, CA). Concentration-response curves were analysed using a three-parameter non-linear regression analysis. Unpaired Student’s t-test was applied for the comparisons of two datasets. One-way or Two-way analysis of variance (ANOVA) with post-hoc test was used where more than two datasets or groups were compared. Statistical significance was accepted at p < 0.05.

## Electronic supplementary material

Supplementary Information
