# Supplementary material for: A tricyclic antidepressant, amoxapine, reduces amyloid-β generation through multiple serotonin receptor 6-mediated targets
Source: Sci Rep. 2017 Jul 10;7:4983. doi: 10.1038/s41598-017-04144-3 (PMC5504036; doi:10.1038/s41598-017-04144-3)

A tricyclic antidepressant, amoxapine, reduces amyloid-β generation through multiple serotonin receptor 6-mediated targets

Xiaohang Li [1,¶], Qinying Wang [1,¶], Tingting Hu [1], Ying Wang [2], Jian Zhao [2], Jing Lu [1,*], and Gang Pei [1,3,*]

[1] State Key Laboratory of Cell Biology, CAS Center for Excellence in Molecular Cell Science, Institute of Biochemistry and Cell Biology, Chinese Academy of Sciences; University of Chinese Academy of Sciences, 320 Yueyang Road, Shanghai 200031, China

[2] Translational Medical Center for Stem Cell Therapy, Shanghai East Hospital, School of Medicine, Tongji University, Shanghai, China,

[3] Shanghai Key Laboratory of Signaling and Disease Research, Collaborative Innovation Center for Brain Science, School of Life Sciences and Technology, Tongji University, Shanghai 200092, China.

* To whom correspondence should be addressed: lu.jing@sibcb.ac.cn; gpei@sibs.ac.cn.

[¶] These authors contribute equally to this work.

Supplementary Table 1. Sequences of primers used for sub-cloning

Plasmids	Identification	Sequence (5'-3')
HTR6-FUGW	Sense	CGTTTTTGGCTTTTTTGTTAGACAGGATCCATG GTCCCAGAGCCGGGC
	Antisense	CATGGTGGCGACCGGTACCCGGACGTTCGTGG GGATGCCAAG
F20-FUGW	Sense	CGTTTTTGGCTTTTTTGTTAGACAGGATCCATG CCTGGCGCAGTAGGC
	Antisense	CATGGTGGCGACCGGTACCCGGTTAAACCCCT TCTGGAGTGATC

Supplementary Table 2. Targeting sequences of shRNAs used for gene silencing

Target	Identification	Sequences 5'-3'
HTR6	shHTR6-1:	GCGCAACACGTCCAACTTCTT
	shHTR6-2:	GCTGCGCTACAAGCTGCGCAT
ARRB	shARRB2-1	CTTCAAGAAGTCGAGCCCTAA
	shARRB2-2	GCTAAATCACTAGAAGAGAAA
CDK5	shCDK5-1	GTGAACGTCGTGCCCAAACTC
	shCDK5-2	CCGGGAGATCTGCCTACTCAA
Gαs	shGαs	CGATGTGACTGCCATCATC
HTR2B	shHTR2B-1	CCGATATATCACCTGCAATTA
	shHTR2B-2	TGCCATTCCAGTCCCTATTAA
HTR4	shHTR4	TGCCATTCCAGTCCCTATTAA
HTR7	shHTR7	TCACCTTACCTCCACTCTTTG,
DRD2	ShDRD2	CACCACCTTCAACATTGAGTT
ARRB1	shARRB1-	AGATCTCAGTGCGCCAGTATG
	shARRB2-	TCTGGATAAGGAGATCTATTA

Supplementary Table 3. Sequences of primers used for RT-PCR and real-time PCR

Gene	Identification	Sequence (5'-3')
HTR2A	Sense	CTTTGTGCAGTCTGGATTTACCT
	Antisense	ACTGATATGGTCCAAACAGCAAT
HTR2B	Sense	TGATTTGCTGGTTGGATTGTTTG
	Antisense	ATGGATGCGGTTGAAAAGAGAA
HTR2C	Sense	TCAATACCTCCGATGGTGGAC
	Antisense	GGTGGCATTGTGCAGTTTCTT
HTR4	Sense	GATCTGCTGGTTTCGGTGCT
	Antisense	CAGAGGGGTCATCTTGTTCCTA
HTR6	Sense	GCAACACGTCCAACTTCTTCC
	Antisense	TGCAGCACATCACGTCGAA
HTR7	Sense	AACAGATCAACTACGGCAGAGT
	Antisense	CACACGGAGATCACCACCA
DRD2	Sense	CTCTTCGGACTCAATAACGCAG
	Antisense	GACGATGGAGGAGTAGACCAC
DRD3	Sense	AGAAGGCAACCCAAATGGTGG
	Antisense	TGTCGTGGCACTGTAAAGCTC
DRD4	Sense	GGTGTCGGACCCTACTCAG
	Antisense	GGCAGGACTCTCATTGCCTT
HPRT	Sense	CCTGGCGTCGTGATTAGTGAT
	Antisense	AGACGTTCAGTCCTGTCCATAA
DCX	Sense	CATCCCCAACACCTCAGAAG
	Antisense	GGAGGTTCCGTTTGCTGA
Tubb3	Sense	GCAACTACGTGGGCGACT
	Antisense	CGAGGCACGTACTTGTGAGA
Map2	Sense	GGGCTGACATCCCACCTA
	Antisense	ATTATTCCACGCTTGCTG
NeuronD1	Sense	CCAGGGTTATGAGACTATCACTG

	Antisense	TCCTGAGAACTGAGACACTCG
SYN1	Sense	CCCCAATCACAAAGAAATGCTC
	Antisense	ATGTCCTGGAAGTCATGCTG
NCAM	Sense	TACCGCGGCAAGAACATC
	Antisense	CCACCTGCAGAGAAACTGC
MAPT	Sense	CCAAGTGTGGCTCATTAGGCA
	Antisense	CCAATCTTCGACTGGACTCTGT
vGluT1	Sense	TCAATAACAGCACGACCCAC
	Antisense	TCCTGGAATCTGAGTGACAATG
ChAT	Sense	GCACTCCAGCTCCTTCAC
	Antisense	CACTGCACCAGGACGATG
TH	Sense	CTGTGGCCTTTGAGGAGAAG
	Antisense	GGTGGATTTTGGCTTCAAAC
vGAD67	Sense	ATGGTGATGGGATATTTTCTCC
	Antisense	GCCATGCCCTTTGTCTTAAC
Sox2	Sense	CTGCTACCCTTGAGACACCTG
	Antisense	GGGCTCTGATCTCTGCATCTAC
Nestin	Sense	CAAGATGCACAACTCGGAGA
	Antisense	CGGGGCCGGTATTTATAATC

Supplementary materials and methods

Aβ40 and Aβ42 measurement

SK-N-SH cells were treated with chemicals at the indicated concentrations for 24 h. The conditioned medium was then collected and subjected to a Human β Amyloid 40 Kit (ExCell Bio) and a Human Aβ42 US ELISA Kit (Life Technologies) for the measurement of Aβ40 and Aβ42 level. The measurement was done according to the manufacturer's guidelines.

Supplementary figure legend

Sup Figure 1. Amoxapine is an Aβ-reducing agent. A. The levels of cell viability of SK-N-SH cells in response to vehicle (0.1% DMSO), 10 μM BSI IV, 10 μM L685,458, or the indicated compounds at 1 μM, 3 μM or 10 μM for 24 hours. B. The levels of Aβ produced by SK-N-SH cells in response to vehicle (0.1% DMSO), 10 μM BSI IV, 10 μM L685,458, or the indicated compounds at 1 μM, 3 μM or 10 μM for 24 hours. C. The levels of cell viability of SK-N-SH cells in response to vehicle (0.1% DMSO), 10 μM BSI IV, 10 μM L685,458, or the indicated compounds at 1 μM, 3 μM or 10 μM for 24 hours. D. The level of Aβ40 or Aβ42 produced by SK-N-SH in response to vehicle (0.1% DMSO) and Amoxapine at 3 μM or 10 μM for 24 hours, and measured by a Human β Amyloid 40 Kit and a Human Aβ42 US ELISA Kit. E. The ratio of extracellular Aβ42/40. Data are presented as the mean ± s.e.m. * $p < 0.05$, ** $p < 0.01$ and *** $p < 0.001$ compared to the control of each group. One-way ANOVA with *post hoc* comparison test (A-C and E) and two-way ANOVA with *post hoc* comparison test (D).

Sup Figure 2. The knockdowns of other major amoxapine binding receptors do not attenuate amoxapine's effect. A. The mRNA expressions of serotonin receptor and dopamine receptor subtypes including HTR2A, HTR2B, HTR2C, HTR4, HTR6, HTR7. DRD2, DRD3 and DRD4 in SK-N-SH cells. B. The mRNA level of DRD2, HTR4 and HTR7 in SK-N-SH cells with the infection of scrambled, DRD2, HTR4, or HTR7 gene specific shRNA. C-E. The levels of Aβ produced by SK-N-SH cells after the treatment with vehicle (0.1% DMSO) or amoxapine at 10 μM for 24 hours in the cells infected as described in (B). Data are presented as the mean ± s.e.m. * $p < 0.05$, ** $p < 0.01$ and *** $p < 0.001$ compared to the control of each group or the control of shNC group. $ $p < 0.05$, $$ $p < 0.01$ and $$$ $p < 0.001$ compared to amoxapine of the shNC group. One-way ANOVA with *post hoc* comparison test (F) and two-way ANOVA with *post hoc* comparison test (B-E).

Sup Figure 3. Differentiated human iPSC derived NSCs characterization. A. The mRNA level of Sox2 and Nestin in human NSCs and NSC differentiated cells. B. The mRNA level of DCX, Tubb3, Map2, NeuroD1, SYN, NCAM, and MAPT in human NSCs and NSC differentiated cells. C. The mRNA level of vGluT1, ChAT, TH and vGAD67 in human NSCs and NSC differentiated cells. Data are presented as the mean ± s.e.m. * $p < 0.05$, ** $p < 0.01$ and *** $p < 0.001$ compared to the control of each group. Two-tailed t-test (A and C), and one-tailed t-test (B).

Sup Figure 4. Uncropped representative image of western blot analysis. A. ADAM10/BACE1/actin expression. B. APP/actin expression. C. β-arrestin2/actin expression. D. CDK5/actin expression.

Sup fig 1. Amoxapine is an Aβ-reducing agent.

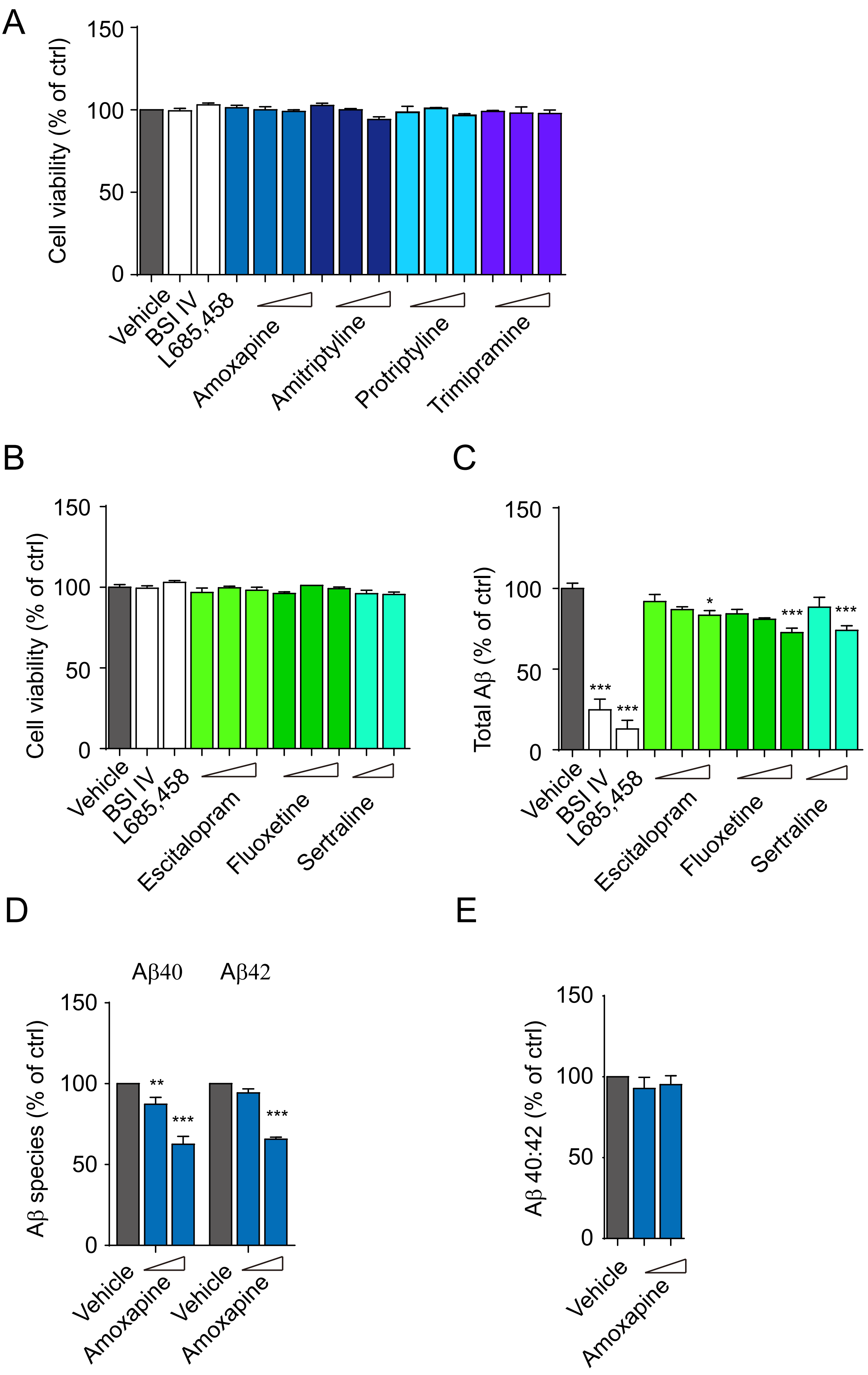

Sup fig 2. The knockdowns of other major amoxapine binding receptors do not attenuate amoxapine's effect.

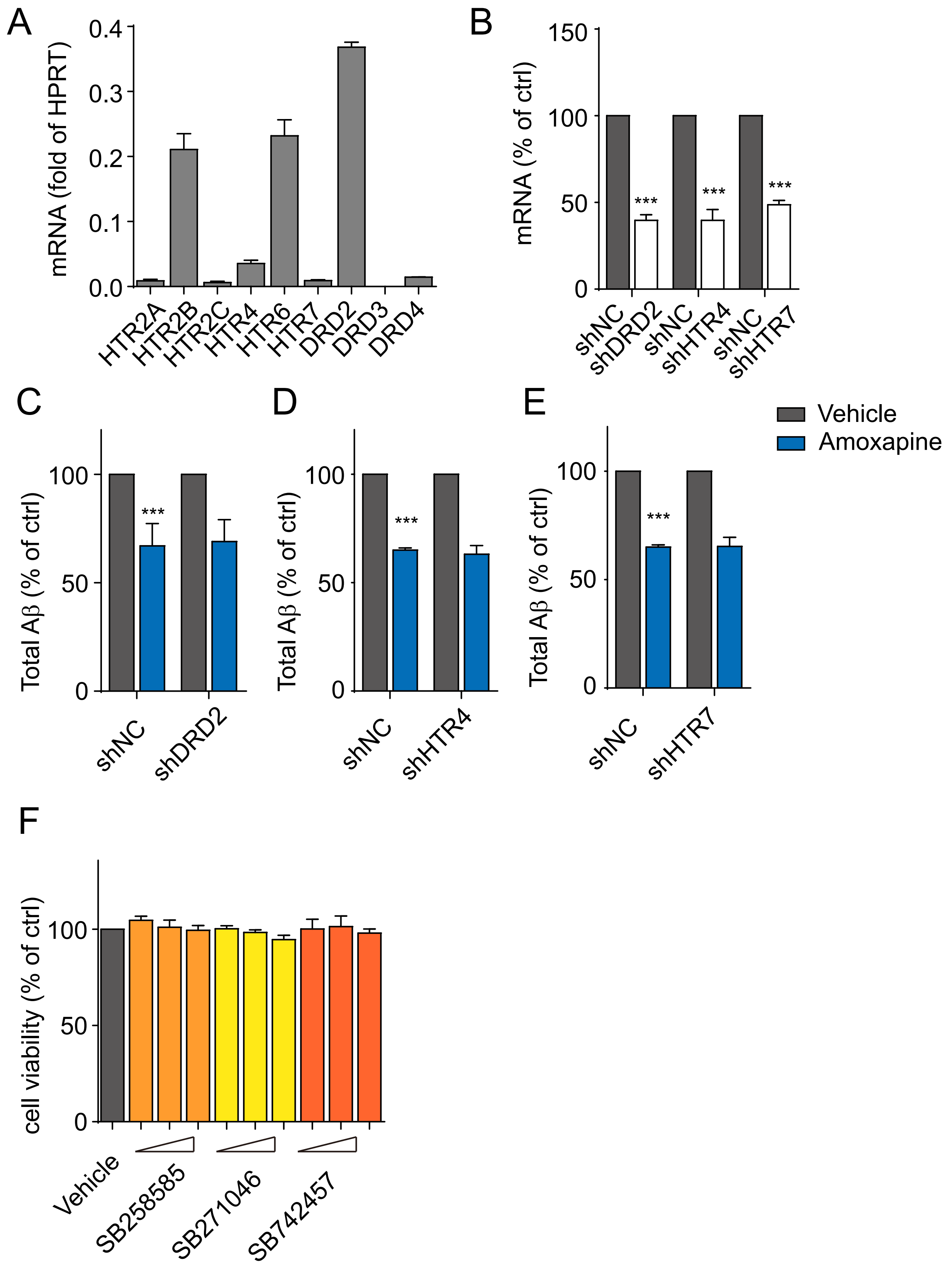

Sup fig 3. Differentiated human iPSC derived NSCs characterization.

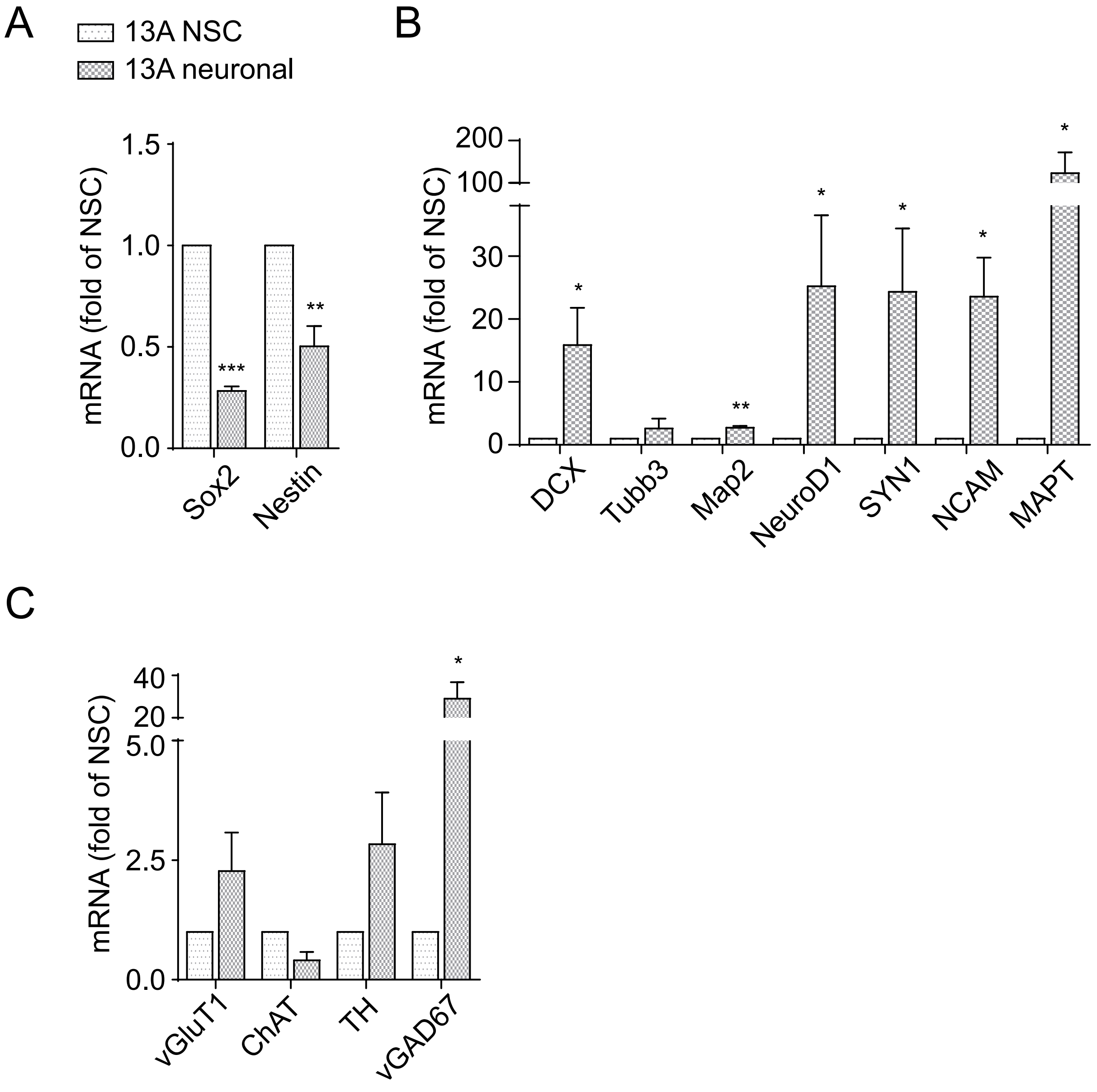

Sup Fig.4 Uncropped image of western blot analysis

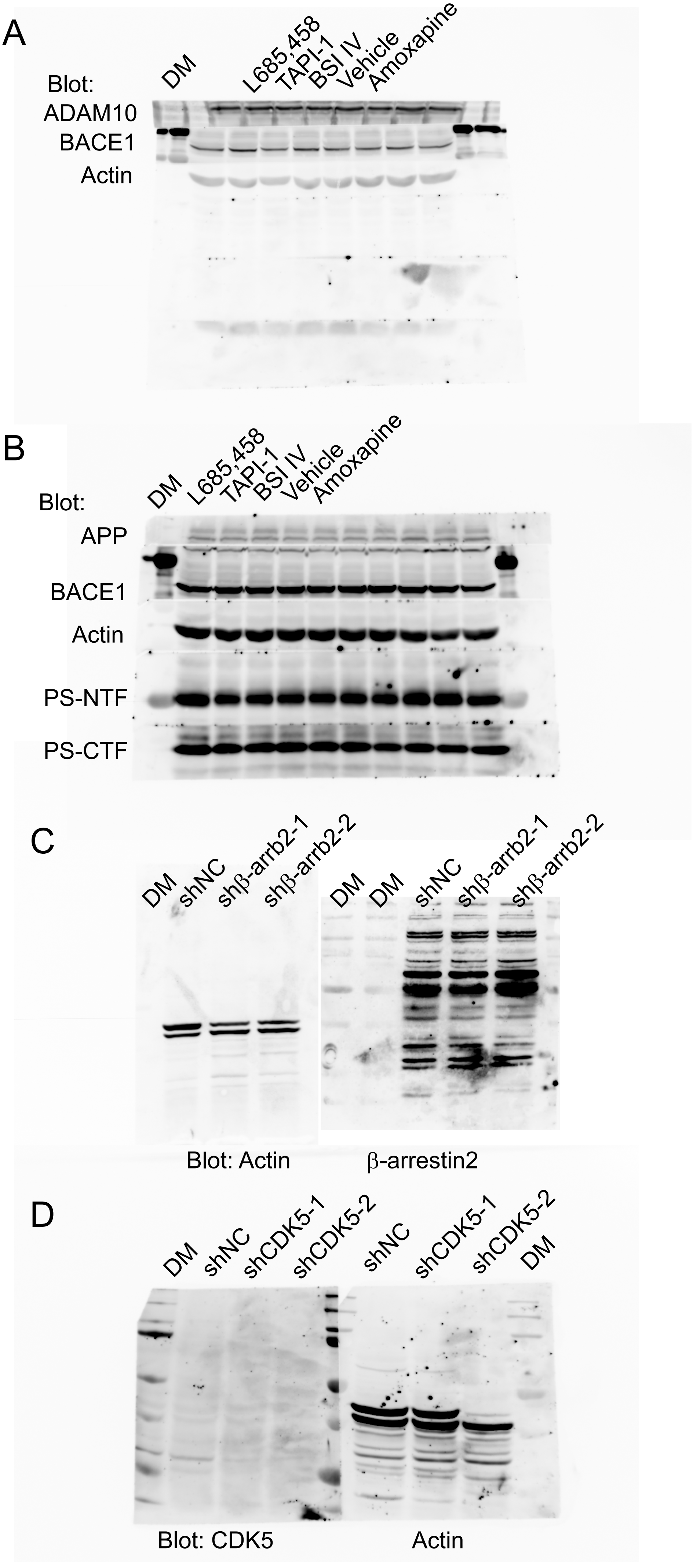

Supplement: Supplementary file 1 — Supplementary Information [file 41598_2017_4144_MOESM1_ESM.pdf]
